# Clonal tracing of rare anal metastasis in esophageal squamous cell carcinoma: a case report with whole-exome sequencing and multimodal therapy

**DOI:** 10.3389/fimmu.2026.1727070

**Published:** 2026-03-26

**Authors:** Yuzhu Jiang, Yu Pu, Zhou Huang, Qin Pei, Mengxia Li, Mingfang Xu

**Affiliations:** 1 Department of Cancer Center, Daping Hospital, Army Medical University, Chongqing, China; 2 Second Affiliated Hospital of Chongqing Medical University, Chongqing, China

**Keywords:** anal metastasis, clonal evolution, esophageal squamous cell carcinoma(ESCC), molecular profiling, whole-exome sequencing

## Abstract

Esophageal squamous cell carcinoma (ESCC) shows considerable variation in incidence across different regions and is affected by various risk factors, including smoking and HPV infection. This article presents a rare case of ESCC that metastasized to the anal region, illustrating the effectiveness of whole-exome sequencing and clonal evolution analysis in identifying the origins of the metastasis. The findings reveal distinct clonal characteristics between the primary tumor and the metastatic site. This underscores the importance of molecular profiling for developing effective treatment strategies.

## Introduction

Esophageal squamous cell carcinoma (ESCC), a common digestive tract malignancy in China, shows significant regional disparities in incidence. Epidemiological studies indicate that the development and progression of ESCC are associated with multiple risk factors, including long-term smoking, alcohol consumption, dietary habits, and genetic susceptibility (1–3). The typical clinical manifestations of this disease include progressive dysphagia, weight loss, and retrosternal pain, with advanced patients often suffering from severe malnutrition due to esophageal obstruction. Meanwhile, anal squamous cell carcinoma, another malignancy with distinct epidemiological characteristics, is closely linked to high-risk human papillomavirus infection, and its incidence has shown an increasing trend in recent years (4, 5).This connection is relevant because, although rare, metastases of ESCC to the anal region have been reported, presenting unique clinical manifestations and therapeutic challenges (6). With the rapid advancement of molecular pathology, significant progress has been made in elucidating the molecular features of ESCC (7–9). Clinical observations have revealed that ESCC may exhibit atypical metastasis, with rectal and anal metastases being rare but drawing considerable attention due to their unique clinical manifestations and therapeutic challenges (10, 11). This metastatic behavior may be associated with various factors, including tumor heterogeneity, microenvironmental characteristics, and host immune status (12–15).

In clinical pathological diagnosis, the differential diagnosis of synchronous esophageal and anal squamous cell carcinomas poses significant challenges. Although modern pathological techniques—such as immunohistochemical detection of markers like p16, CK5/6, and p63—can provide important references, the expression of these markers often overlaps between the two tumor types, limiting their specificity (16–18).

Consequently, molecular testing technologies, such as clonal evolution analysis (e.g., whole-exome sequencing), have demonstrated significant clinical utility in determining the primary tumor site and metastatic relationships of tumors (19).

In this article, we report a rare case of esophageal squamous cell carcinoma (ESCC) with anal metastasis. By integrating clinical diagnosis and comprehensive genomic profiling via whole-exome sequencing (WES), we reveal the clonal evolution pattern of the metastatic lesion. This provides valuable insights for the precise diagnosis and treatment of similar complex metastatic cases.

## Materials and methods

### Mutation calling

Raw sequencing reads were preprocessed using FASTP (v.1.0.1) with default parameters for quality control, including adapter trimming and removal of poly(N) and low-quality reads (20). The cleaned reads were then aligned to the human reference genome (GRCh38/hg38) using BWA (v.0.7.19). Samtools (v.1.22.1) was used to sort the mapped BAM files, and then GATK (v.4.6.2.0) was applied to process PCR duplicates and perform base quality score recalibration (21–23). Somatic mutations, including single-nucleotide variants (SNVs) and small insertions and deletions (indels), were called using Mutect2 (v.4.6.2.0), which was integrated into GATK.

Somatic mutations were called using Mutect2 by comparing each tumor sample with its matched normal sample. Only variants labeled as ‘PASS’ were retained. The resulting variants were further filtered against a BED file defining the WES target regions to exclude off-target calls. Putative germline SNPs were removed using the files from gnomAD database, with the parameter “--af-of-alleles-not-in-resource” set to 0.0000025. In addition, cross-sample contamination was estimated using GATK CalculateContamination, and variants potentially arising from contamination were filtered out based on the estimated contamination level. Maftools (v.2.24.0) was used for the visualization of mutation data (24).

### Mutational signature extraction

We extracted single-base substitution (SBS), doublet-base substitution (DBS), and small insertion and deletion (ID) signatures using SigProfilerExtractor (v.1.2.1) from the SigProfiler tool suite (25). For signature extraction, 1,000 iterations were performed (nmf_replicates = 1000). A signature was considered to be supported by the program if the extracted signature can be reconstructed by multiple SigProfiler-extracted signatures (reconstruction cosine similarity ≥ 0.95). The extracted signatures were compared against the COSMIC v3.2 reference database, and those with a cosine similarity of ≥ 0.95 were assigned as known signatures.

### Copy number variations

CNVkit (v.0.9.12) was used to infer and visualize copy number alterations from WES data (26). Tumor and matched normal BAM files were provided as input to calculate log2 depth ratios, which were normalized using the GC content bias and data ratio. Absolute copy numbers were called using CNVkit’s *call* command, and the heatmap of copy number profiles across multiple samples was generated with the heatmap module. Focal regions of somatic copy number gain and loss were identified using GISTIC2.0 (Genomic Identification of Significant Targets in Cancer) (27). Significant focal aberrations were identified at a confidence threshold of 0.98.

### Inferring clonality and evolution

Clonal architecture and evolutionary trajectories were inferred from somatic variant allele frequencies (VAF). Subclonal architectures were first inferred using PyClone (v.0.13.1) (28), which groups mutations into clusters based on their estimated cellular prevalence. Clonal phylogeny and genotypes were then reconstructed from these clusters using Citup (v.0.1.2) (29), with cellular frequencies derived from whole-exome sequencing of multiple tumor biopsies as input. Temporal clonal dynamics were then visualized with TimeScape (v.1.32.0), illustrating shifts in subclonal abundance and lineage divergence across sequential samples or time points.

## Case presentation

### Basic information

**Figure 1:**
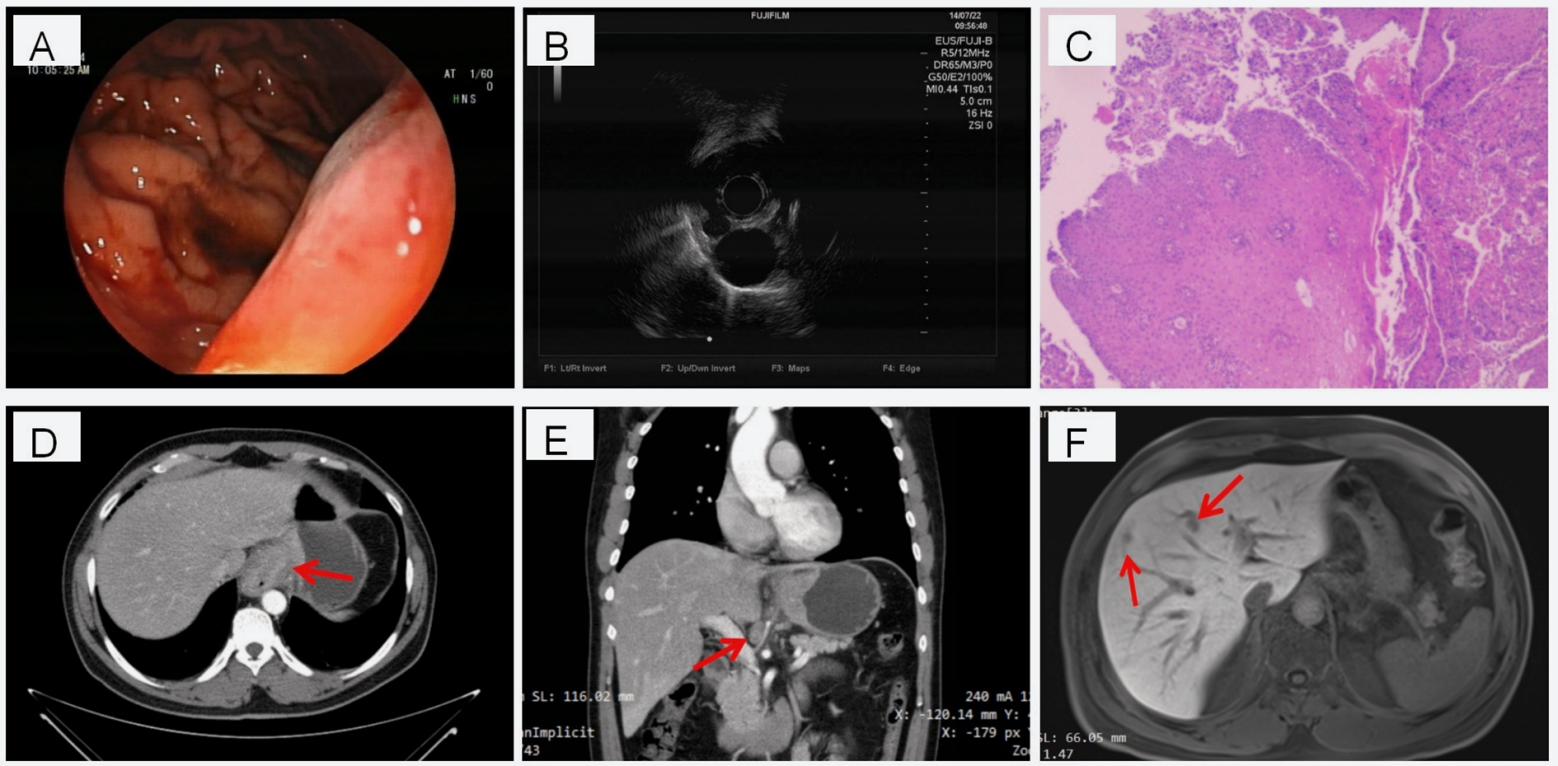
Initial diagnostic imaging and pathology of the primary esophageal squamous cell carcinoma (ESCC). **(A, B)** Endoscopic ultrasonography images showing a neoplastic lesion involving all four walls of the esophagus at 32 cm from the incisors, with invasion into the muscularis propria (T3 stage) and associated peritumoral lymphadenopathy. **(C)** Histopathological examination (H&E stain) of the endoscopic biopsy confirming esophageal squamous cell carcinoma. **(D–F)** Typical enhanced CT images of the chest/abdomen **(D, E)** and an MRI image of the upper abdomen **(F)** show the primary tumor in the lower esophagus, metastatic lymph nodes between the liver and stomach, and liver metastases (indicated by arrows).

A 47-year-old male patient presented in July 2022 with progressive dysphagia for dry, solid foods. Endoscopic ultrasonography (2022–07–01) revealed a neoplastic lesion involving all four walls of the esophagus at 32 cm from the incisors, extending to the subcardiac region. The tumor invaded the muscularis propria, corresponding to stage T3, and multiple enlarged peritumoral lymph nodes were observed (**Figures 1A, B**). Pathological examination confirmed esophageal squamous cell carcinoma (**Figure 1C**). Contrast-enhanced CT of the chest and abdomen, along with upper abdominal MRI, further identified: (1) a space-occupying lesion in the lower esophagus and cardia; (2) metastatic lymphadenopathy in the mediastinum and hepatogastric space (cN2); (3) multiple hypoenhancing hepatic nodules suspected to be metastases (**Figures 1D–F**). Based on these imaging and pathological findings, the final diagnosis was gastroesophageal-junction squamous cell carcinoma (cT3N2M1, Stage IV) with liver metastases. The patient had no family history of cancer in first-degree relatives. He had a 15-year smoking history (20 cigarettes/day) until quitting in 2014, and a >20-year history of alcohol consumption (500 mL liquor daily) until cessation upon cancer diagnosis in 2022.

### Treatment course and clinical outcomes

**Figure 2:**
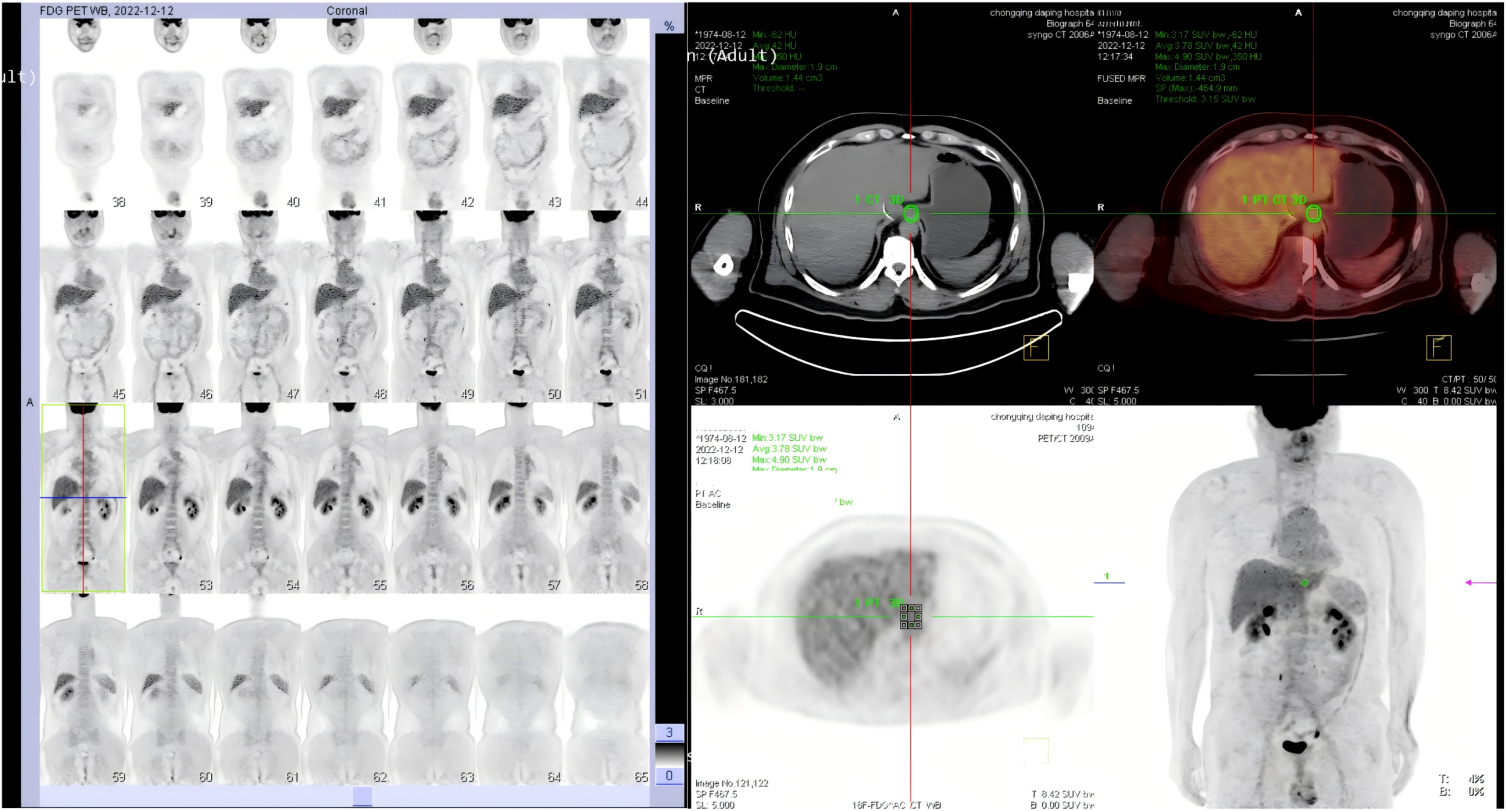
Treatment response assessment. **(A, B)** Fused PET-CT images at the level of the esophagogastric junction before **(A)** and after **(B)** neoadjuvant therapy, showing significant metabolic regression of the primary tumor (SUVmax reduced) and complete resolution of previously noted liver metastases, indicating a complete clinical response (CR).

**Figure 3:**
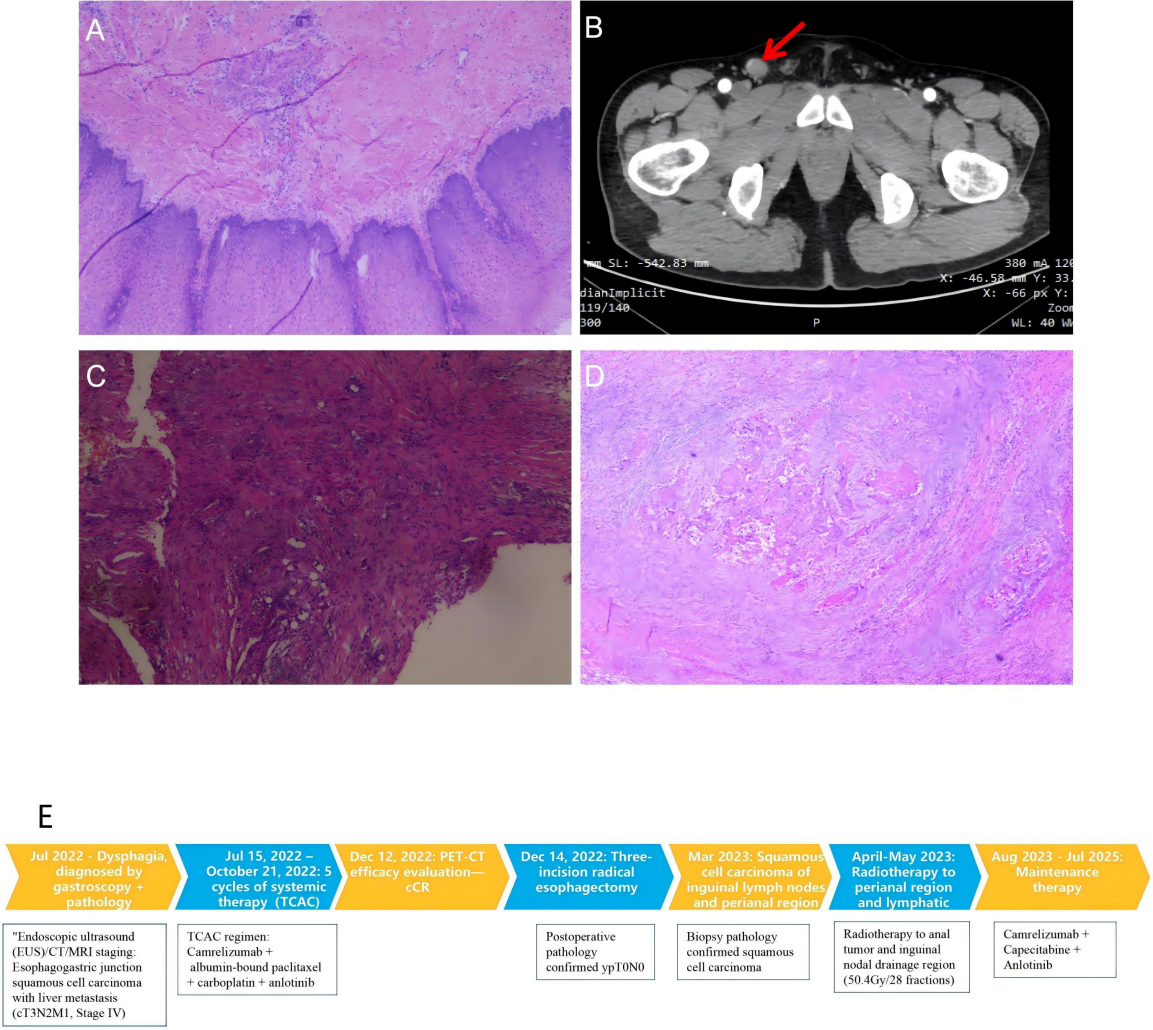
Postoperative pathology and flowchart. **(A)** Histopathological image (H&E stain) of the surgical esophagectomy specimen post-neoadjuvant therapy, showing no residual viable tumor cells (ypT0), consistent with a pathological complete response (pCR). **(B)** Contrast-enhanced CT of the pelvis revealing an anal canal mass (arrow). **(C, D)** Biopsy specimens from the anal lesion **(C)** and an inguinal lymph node **(D)** (H&E stain), both confirming squamous cell carcinoma. **(E)** Timeline of Diagnosis, Treatment, and Clinical Course. Schematic overview illustrating the key events in the patient’s management from initial presentation (July 2022) to the last follow-up (February 2026). This includes the timeline of: initial diagnosis of stage IV ESCC; neoadjuvant combination therapy with camrelizumab, albumin-bound paclitaxel, carboplatin, and anlotinib; radical esophagectomy; maintenance therapy; subsequent diagnosis of anal and inguinal lymph node metastases (March 2023); and subsequent treatment with radiotherapy and adjusted systemic therapy.

After enrollment in clinical trial NCT04471480, the patient received five cycles of systemic chemotherapy from July 15, 2022, to October 21, 2022, consisting of camrelizumab 200 mg IV on day 1, albumin-bound paclitaxel 400 mg IV on day 1, carboplatin 550 mg IV on day 1, and anlotinib 8 mg orally from day 1 to 14. The tumor showed significant regression; PET-CT at 5 months demonstrated only mild mucosal thickening at the esophagogastric junction with low FDG uptake and complete resolution of liver metastases, thereby achieving a complete clinical response (CR) (**Figures 2A, B**). Subsequently, the patient underwent radical esophagectomy with three-field lymphadenectomy on December 14, 2022. Postoperative pathology confirmed ypT0N0 (CAP grade 0) with no metastases in all 27 resected lymph nodes (**Figure 3A**). Maintenance therapy with camrelizumab plus anlotinib was continued thereafter.

In March 2023, enhanced CT and biopsy confirmed squamous cell carcinoma of the anus along with metastatic squamous cell carcinoma in bilateral inguinal lymph nodes (**Figures 3B–D**). The patient received radiotherapy of 50.4 Gy in 28 fractions to the anal region and inguinal nodal basin, with adjusted systemic therapy consisting of camrelizumab, capecitabine, and anlotinib. The patient is expected to remain disease-free as of February 2026. The diagnostic and therapeutic timeline for the patient is presented in (**Figure 3E**).

### Molecular characterization and analysis

**Figure 4:**
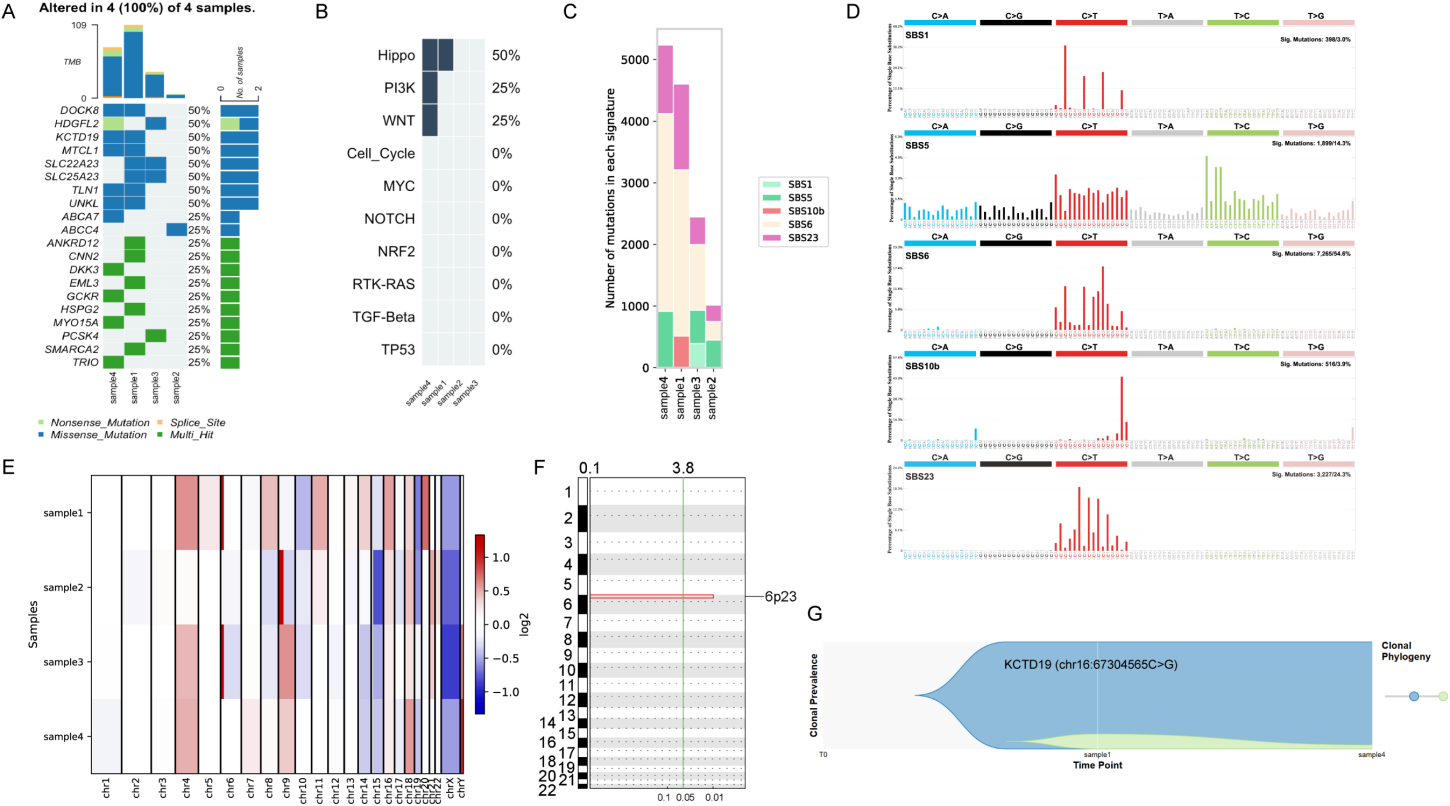
Comprehensive genomic profiling and clonal evolution analysis. **(A)** Oncoplot depicting the landscape of recurrent somatic mutations (e.g., in DOCK8, HDGFL2, KCTD19) across the four analyzed samples: primary ESCC (Sample 1), post-treatment pCR tissue (Sample 2), inguinal lymph node metastasis (Sample 3), and anal metastasis (Sample 4). Mutation types are color-coded (e.g., missense in green, nonsense in red). **(B)** Plot illustrates which pathways were affected by the mutated genes. **(C, D)** Plots showing the contributions of different mutational signatures **(C)** and their consistency **(D)** across the four samples, supporting clonal relatedness while highlighting evolutionary divergence. **(E)** Heatmap of genome-wide copy number variation (CNV) profiles. Samples 1, 3, and 4 show comparable patterns of chromosomal gains and losses, while Sample 2 (pCR) shows a profile similar to normal tissue. **(F)** The specific high-level amplification at the 6p23 locus (log2 ratio = 3.8) in Samples 1 and 3 is highlighted. **(G)** Schematic representation of the phylogenetic tree inferred from shared mutations, illustrating the clonal evolutionary relationship between the primary tumor and its metastatic sites. The identical KCTD19 mutation (chr16:67304565C>G) is indicated in the fishplot to illustrate its phylogenetic placement and support the inferred clonal relationship between Sample 1 and Sample 4.

Whole-exome sequencing (WES) and comprehensive genomic profiling were conducted on four patient samples: pretreatment esophageal squamous cell carcinoma (Sample 1), post-neoadjuvant therapy pathological complete response (pCR) esophageal tissue (Sample 2), postoperatively identified anal squamous cell carcinoma (Sample 4), and synchronously detected metastatic squamous cell carcinoma in the inguinal lymph node (Sample 3). The analysis of genomic variations focused on tumor mutational profiles, mutational pathways, mutational signatures, copy number variations (CNVs), and tumor clonal evolution to clarify clonal origins and metastatic progression, as illustrated in **Figure 4** (datasets are available in CNCB and the accession numbers are PRJCA048316).

The CNV analysis revealed a significant high-level amplification at the 6p23 locus (log2 ratio = 3.8) in Sample 1, the primary esophageal tumor, which was also present in Sample 3, the lymph node metastasis, indicating a shared clonal origin (**Figures 4E, F**). In contrast, Sample 2, the pCR tissue, exhibited no significant amplification, with CNV levels similar to those of normal tissue (log2 ratio range: -1 to 1). The CNV heatmap further supported this observation, showing that Samples 1, 3, and 4 had comparable global CNV profiles characterized by gains and losses across various chromosomal regions, while Sample 2 displayed a minimal CNV burden, reinforcing the molecular basis for the pathological complete response (**Figure 4E**).

The analysis of the tumor mutational landscape revealed recurrent alterations in genes such as DOCK8, HDGFL2, and KCTD19 in both Sample 1 and Sample 4, as illustrated in **Figure 4A**. The classification of variants showed that missense mutations, indicated by green bars, and nonsense mutations, indicated by red bars, were the most common types. Oncogenic signaling pathways affected by mutated genes in each sample are shown in **Figure 4B**. Notably, the same KCTD19 mutation (chr16:67304565C>G) was detected in Sample 1 and Sample 4 with different VAFs, which indicates a clonal relationship between two samples. Clonal architecture reconstruction and evolutionary trajectory analysis indicated that Sample 4 arose from metastatic seeding of the subclone carrying this mutation, which disseminated from the primary esophageal tumor to the perianal region at an early stage and subsequently established successful colonization (**Figure 4G**). By contrast, Sample 3, a metastatic tumor in the lymph node, and Sample 4, an anal squamous cell carcinoma, exhibited unique private mutations, indicating that clonal evolution was actively occurring during the metastatic process.

The analysis of mutational signatures demonstrated a high degree of consistency across all four samples, reinforcing their clonal relatedness. However, variations in the contribution of these signatures pointed to clonal heterogeneity as the evolution progressed (**Figure 4C, D**).

Furthermore, multidimensional genomic profiling illustrated a hierarchical accumulation of genetic changes. Sample 1, the primary tumor, displayed foundational clonal features, such as amplification of chromosome 4, which were also present in Samples 3 and 4. However, Sample 4 acquired additional subclonal amplifications in chromosomes 9 and 18, reflecting ongoing clonal evolution. This observation supports the “seed and soil” theory of metastasis, which posits that metastatic subclones adapt and evolve within different microenvironments, as evidenced by the distinct subclonal amplifications in Sample 4. Overall, the shared patterns of copy number alterations, the conserved mutational signatures, and the hierarchical accumulation of genetic alterations provide strong evidence that the anal squamous cell carcinoma (SCC) in Sample 4 and the inguinal lymph node metastasis in Sample 3 originated from the primary esophageal SCC in Sample 1. These findings indicate that they did not arise as independent primary tumors.

## Discussion

Esophageal squamous cell carcinoma, as a highly aggressive malignant tumor, presents significant clinical challenges due to its complex metastatic patterns and marked tumor heterogeneity (30). The present case of ESCC with anal metastasis is extremely rare in clinical practice. Diagnostic difficulties arise from the limitations of traditional histomorphology and immunohistochemistry in distinguishing metastatic lesions from multiple primary tumors (6). This study, through multimodal clinical diagnostics and WES-based genomic profiling, provides the first evidence of shared driver mutations between the primary esophageal tumor and the anal metastatic lesion, offering new insights into the metastatic biology of ESCC.

Building on these findings, whole-exome sequencing and clonal evolution analysis in this patient not only confirmed the anal lesion’s origin from the primary esophageal tumor but also revealed a unique metastatic pattern. The lack of 6p23 amplification in the anal metastasis—despite its clonal presence in the primary tumor and lymph node metastases—suggests independent metastatic seeding rather than sequential spread. However, multi-region sequencing of the anal lesion would be required to rule out intratumoral heterogeneity or sampling limitations, which represents a limitation of the current study. Further analysis demonstrated that the inguinal lymph node metastases retained the primary tumor’s clonal characteristics (chromosome 6p and 18 amplification) while acquiring amplifications on chromosomes 7q and 20q through clonal evolution. This finding aligns with the “seed and soil” theory of tumor metastasis and may explain the more aggressive behavior of the inguinal lymph node metastases.

The molecular analysis in this study highlights the complexity of ESCC metastasis. While both the anal lesion and inguinal lymph node metastases originated from the primary esophageal tumor, they exhibited distinct clonal features. The anal metastasis’s retention of ancestral mutations (e.g., KCTD19) but loss of 6p23 amplification may reflect early dissemination or result from selective pressure in the anal microenvironment. Single-cell sequencing of primary and metastatic lesions could clarify the timing and route of spread. The dynamic changes in copy number variation (CNV) from the primary lesion, the surgical specimen, and the recurrent lesion indicate that the tumor develops drug resistance through the selection and acquisition of new variants under treatment pressure (31). These findings provide critical implications for clinical decision-making. First, this case suggests that molecular profiling may aid in the differential diagnosis of rare metastases, though its clinical value requires validation in prospective cohorts. Second, it suggests the potential for individualized treatment strategies based on distinct molecular features of different metastatic sites (32, 33). Third, it confirms the crucial role of multidisciplinary collaboration in managing advanced ESCC (34).

The therapeutic implications from this case extend beyond its rarity. The patient, diagnosed with T3N2M1 (Stage IV) ESCC and liver metastases, achieved a pathological complete response (pCR) following combination therapy with camrelizumab, albumin-bound paclitaxel, carboplatin, and anlotinib. This regimen aligns with recent reports on the efficacy of small-molecule tyrosine kinase inhibitors (TKIs) combined with immunotherapy in advanced ESCC (35–37). Importantly, the patient’s tumor carries a DOCK8 mutation. While DOCK8 mutations have been associated with immune-rich microenvironments in other cancers (38), the functional significance of this specific variant remains unclear. Future studies should correlate such mutations with PD-L1 expression or T-cell infiltration in ESCC to assess their predictive value for response to immunotherapy.

While the combination therapy used in this case demonstrated remarkable efficacy, its broader applicability requires careful consideration. First, the cost and availability of immune checkpoint inhibitors and targeted therapies may limit their use in settings with limited resources, such as low-income countries or underfunded hospitals. Future studies should explore cost-effective alternatives or biomarker-driven patient selection to optimize resource allocation. Second, the patient’s unique molecular profile, specifically the identified genetic alterations, raises questions about whether these alterations should be routinely screened to guide therapy in advanced ESCC (39).

Study limitations include the unavailability of liver metastasis tissue samples for molecular validation and the need for further investigation into anlotinib’s specific role in the combination therapy. To address these limitations, future research should focus on (1): developing clonal evolution-based models to predict metastatic risk and guide surveillance protocols (2); designing clinical trials to evaluate the generalizability of this combination therapy in unselected ESCC populations; and (3) exploring metastasis-specific genetic alterations as therapeutic targets.

## Patient perspective

My journey with esophageal squamous cell carcinoma began in July 2022, when I was diagnosed with stage IV disease and liver metastases. The news was devastating. However, being offered enrollment in a clinical trial provided a crucial glimmer of hope. I agreed to proceed with the novel combination therapy of camrelizumab, chemotherapy, and anlotinib.

The most encouraging part of my experience was the dramatic response to initial treatment. Within months, follow-up scans showed the primary tumor had significantly shrunk and the liver metastases had completely disappeared—a result far exceeding my expectations. This gave me immense confidence in the treatment plan and medical team. Although surgery was not initially recommended due to the metastases, I strongly advocated for and ultimately underwent esophagectomy in pursuit of a potential cure, a decision later supported by the pathological confirmation of a complete response.

The discovery of a new mass in the anal region in March 2023 was a profound psychological setback, raising fears of treatment failure. At this critical point, the rapid multi-disciplinary evaluation and advanced molecular testing by my medical team were essential. Confirming that this was a metastasis from my original cancer—not a new primary tumor—helped me reframe this recurrence as a continuation of the same battle, requiring a strategic adjustment rather than starting over.

Subsequent pelvic radiotherapy and adjustments to systemic therapy required resilience. The integration of local control with continued systemic treatment, tailored based on the unique molecular features of my cancer, gave me a strong sense of personalized care.

As of my last follow-up, maintaining a disease-free status remains my focus. This entire experience has highlighted for me the vital importance of access to advanced diagnostics, innovative therapies, and, above all, a cohesive, multi-specialty medical team that expertly navigates both the clinical complexities and the human dimensions of this disease. I hope the molecular insights from my case can contribute to better strategies for future patients facing similar rare metastatic challenges.

## Conclusion

This case study, through the integration of clinical response and molecular characterization, reveals the complex biological behavior of ESCC metastasis. It also demonstrates the critical importance of multidisciplinary collaboration and molecularly guided strategies in managing advanced ESCC. Genomic instability and clonal evolution emerge as key determinants of treatment response and metastatic patterns. While the reported combination therapy shows promise, its widespread adoption requires further validation of predictive biomarkers and cost-effectiveness analyses. These findings underscore the need for personalized therapeutic approaches and robust long-term monitoring to improve outcomes in advanced ESCC.

## Data Availability

The datasets presented in this study can be found in online repositories. The names of the repository/repositories and accession number(s) can be found below: https://ngdc.cncb.ac.cn, PRJCA048316.
